# Levels of Physical Activity among Participants in the JACC Study

**DOI:** 10.2188/jea.15.S43

**Published:** 2005-05-16

**Authors:** Youichi Kurozawa, Takenobu Hosoda, Nobuo Iwai, Takayuki Nose, Takesumi Yoshimura, Akiko Tamakoshi

**Affiliations:** 1 Division of Health Administration and Promotion, Department of Social Medicine, Faculty of Medicine, Tottori University.; 2 Chugoku Occupational Health Association.; 3 Fukuoka Institute of Health and Environmental Sciences.; 4 Department of Preventive Medicine/Biostatistics and Medical decision Making, Nagoya University Graduate School of Medicine.

**Keywords:** JACC Study, physical activity, habitual physical exercise, walking

## Abstract

BACKGROUND: Physical activity is thought to play an important role in the maintenance and promotion of health, and practical questionnaires assessing levels of physical activity are currently a widely used method in epidemiological research.

METHODS: As a part of the Japan Collaborative Cohort Study (JACC Study) for Evaluation of Cancer Risk sponsored by the Ministry of Education, Science, Sports and Culture of Japan (Monbusho), we investigated the status of physical activity of cohort participants aged 40-79. A total of 110,792 participants (46,465 men and 64,327 women) completed the baseline survey from 1988-1990. Questionnaires concerning physical activity contained the following questions: 1) How much time per week on average do you spend engaging in sports or physical exercise? (at least 5 hours, 3-4 hours, 1-2 hours, little); 2) How much time per day on average do you spend walking either indoors or outdoors? (longer than 1 hour, 30 min-1 hour, about 30 min, little).

RESULTS: In total, 68.7% of men and 76.2% of women responded with “little” to the amount of time spent engaged in sports or physical exercise. The proportion of time spent on habitual physical exercise (sports and physical exercise of one hour or more per week) was lowest in the 50-59 years age group and increased with age among participants aged 50-79. In addition, 49.4% of men and 50.7% of women responded with “more than 1 hour per day” to the amount of time spent walking.

CONCLUSION: These results show that the proportion of habitual physical exercise was relatively low in the JACC Study and increased with age among participants aged 50-79, whereas almost half walked for more than one hour a day.

Physical activity is thought to play an important role in the maintenance and promotion of health, and practical questionnaires assessing levels of physical activity are currently a widely used method in epidemiological research.^1^ The Japan Collaborative Cohort Study (JACC Study) for Evaluation of Cancer Risk sponsored by the Ministry of Education, Science, Sports and Culture of Japan (Monbusho) begun in 1988 to evaluate cancer risk modification as a result of changes in lifestyle.^2^ The JACC Study questionnaires include single-item questions concerning physical activity. In this study, we describe the status of physical activity of participants in the JACC Study.

## METHODS

### Subjects

As part of the JACC Study, a cohort of 110,792 participants (46,465 men and 64,327 women) aged 40 to 79 years completed a baseline survey from 1988 through 1990. Full details of the methodology for establishing the JACC Study is described elsewhere.^2^ In brief, the JACC study is a nationwide collaborative multi-institute study designed to investigate the relationship between risk or protective factors, such as past medical history, family medical history, smoking, alcohol consumption, dietary habits, physical activity, and occupation, with death from selected cancers. Twenty-four institutes took part in the JACC Study and cohort participants were recruited from 45 areas within 19 prefectures in Japan.

### Questionnaire and Data Correction

After obtaining informed consent for participation in the study, subjects were either interviewed on the questionnaire content or filled in the questionnaire themselves. Self-administered questionnaires included information on the subjects’ past and family medical history, general health and lifestyle habits, for example, smoking, drinking, diet, and physical activity habits, and occupation.

The questionnaire concerning physical activity contained the following:

Question 1: How much time per week on average do you spend engaging in sports or physical exercise? (at least 5 hours, 3-4 hours, 1-2 hours, little). In the present study, habitual physical exercise was defined as sports or physical exercise of one hour or more per week.
Question 2: How much time per day on average do you spend walking either indoors or outdoors? (longer than 1 hour, 30 min-1 hour, about 30 min, little).

### Statistical Analysis

SAS^®^ version 8.2 software (SAS institute, Cary, North Carolina, USA) was used for statistical analysis. All results were considered significant at a 5 % critical level.

## RESULTS

Table 1 shows the distribution of responses to Question 1 (time spent on sports or physical exercise) by sex and age group. 37,321 men and 51,135 women replied to Question 1. In total, 68.7% of men and 76.2% of women responded with “little”. The proportion of time spent on habitual physical exercise was lowest in the 50-59 years age group and increased with age among participants aged 50-79.

**Table 1.** Distributions of the responses to Question 1 (time spent on sports or physical exercise) by sex and age.

	Age (year)				total

	40-49	50-59	60-69	70-79	
Men					
5+ hours/week	384 (3.9)	569 (5.1)	1047 (9.5)	721 (14.1)	2721 (7.3)
3-4 hours/week	657 (6.7)	608 (5.4)	832 (7.5)	618 (12.1)	2715 (7.2)
1-2 hours/week	2068 (21.0)	1622 (14.4)	1675 (15.1)	914 (17.9)	6279 (16.8)
little	6763 (68.4)	8466 (75.1)	7530 (67.9)	2847 (55.9)	25606 (68.7)

Women					
5+ hours/week	279 (2.2)	562 (3.5)	903 (5.9)	617 (9.2)	2361 (4.6)
3-4 hours/week	579 (4.5)	678 (4.2)	991 (6.5)	537 (8.0)	2785 (5.4)
1-2 hours/week	1900 (14.7)	2055 (12.8)	2170 (14.1)	925 (13.8)	7050 (13.8)
little	10187 (78.6)	12803 (79.5)	11309 (73.5)	4640 (69.0)	38939 (76.2)

Percentages in parentheses.

Table 2 shows the distribution of responses to Question 2 (time spent on daily walking) by sex and age group. 35,436 men and 49,316 women replied to Question 2. In total, 49.4% of men and 50.7% of women responded with “more than 1 hour”. No difference in daily walking time was found between men and women. Overall, 9.2 to 14.8% of participants spent little time walking per day and this proportion tended to decrease with age.

**Table 2.** Distributions of the responses to Question 2 (time spent walking) by sex and age.

	Age (year)				total

	40-49	50-59	60-69	70-79	
Men					
1+ hour/day	4270 (45.8)	5444 (51.3)	5370 (50.9)	2371 (47.6)	17455 (49.4)
30min- 1 hour/day	1820 (19.5)	1913 (18.0)	2056 (19.5)	1115 (22.4)	6904 (19.4)
about 30 min/day	1854 (19.9)	1851 (17.5)	1944 (18.5)	972 (19.5)	6621 (18.6)
little	1374 (14.8)	1394 (13.2)	1166 (11.1)	522 (10.5)	4456 (12.6)

Women					
1+ hour/day	6051 (50.1)	7813 (52.2)	7785 (51.9)	3234 (48.1)	24883 (50.7)
30min- 1 hour/day	2443 (20.2)	2983 (19.9)	3134 (20.9)	1471 (21.9)	10031 (20.4)
about 30 min/day	2081 (17.2)	2470 (16.5)	2695 (18.0)	1352 (20.1)	8959 (18.2)
little	1511 (12.5)	1701 (11.4)	1379 (9.2)	666 (9.9)	5257 (10.7)

Percentages in parentheses.

Table 3 shows the relationship between the responses to Questions 1 and 2. Spearman’s rank correlation coefficients were 0.166 and 0.140 for men and women, respectively, showing a significant but low correlation. The proportions of participants who reported little time spent on physical exercise and walking were 11.9% in men and 11.0% in women.

**Table 3.** Relationship between responses to Questions 1 (time spent on sports or physical exercise) and 2 (time spent walking)

Q1: Sports and physical exercise	Q2:Daily walking time			

	1+ hour/day	30min-1 hour/day	about 30 min/day	little
Men				
5+ hours/week	2106 (5.4)	454 (1.2)	259 (0.7)	56 (0.1)
3-4 hours/week	1319 (3.4)	837 (2.2)	533 (1.4)	106 (0.3)
1-2 hours/week	3001 (7.7)	1810 (4.6)	1741 (4.5)	412 (1.1)
little	12190 (31.1)	4455 (11.4)	5083 (13.0)	4653 (11.9)

Women				
5+ hours/week	1818 (3.5)	332 (0.6)	148 (0.3)	80 (0.2)
3-4 hours/week	1525 (2.9)	693 (1.3)	435 (0.8)	101 (0.2)
1-2 hours/week	3642 (7.0)	1980 (3.8)	1664 (3.2)	369 (0.7)
little	18713 (35.8)	7667 (14.7)	7328 (14.0)	5725 (11.0)

Percentages in parentheses.

## DISCUSSION

In this study, two thirds of men and three fourths of women responded with “little” to the question concerning time spent on sports or physical exercise. The proportions of participants who spent time on habitual physical exercise ranged from 24.9 to 44.1% in men and 20.5 to 30.9% in women according to age group.

**Figure1.**
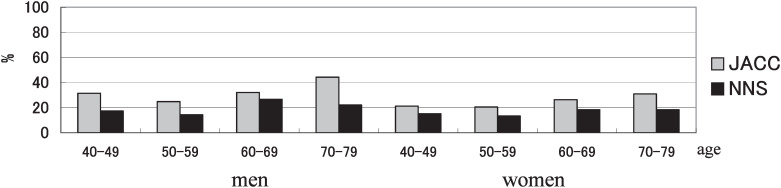
Prevalence of habitual physical exercise by sex and age in the baseline survey of the JACC Study and National Nutrition Survey in Japan (1989).

As shown in Figure 1, we compared these results with the data of the National Nutrition Survey conducted in 1989.^3^ In the National Nutrition Survey, the methods including the definition of habitual physical exercise (physical exercise of 30 min or more on two days or more per week over one year) were different from those in the JACC Study. The trends with age were similar in both surveys. The prevalence of habitual physical exercise was lowest in the 50-59 year age group and increased with age among participants aged 50-79. The increase may be related to increase of leisure time due to retirement. The prevalence of habitual physical exercise in the JACC Study was higher than that in the National Nutrition Survey. Our cohort population was not established by random sampling as performed in the National Nutrition Survey. The cohort members were recruited from volunteers who attended to screening programs for chronic diseases in the 45 areas throughout Japan.^2^ It is well known that there are many differences in the characteristics of those who participate in screening or other health program and those who do not. The participants in the JACC Study are thought to be healthier than the general population and to be more likely to comply with medical recommendations. It may explain the difference of prevalence of habitual physical exercise between the two surveys.

The Japan Lifestyle Monitoring Study (JLM Study)^4^ described leisure-time physical activity status among middle-aged Japanese individuals. Our results were relatively consistent with those of the Japan Lifestyle Monitoring Study, showing that the proportion of individuals who spent time on habitual physical activity (active cases) ranged from 16 to 59 % according to sex and region.^4^

Almost half the participants in the JACC Study walked for more than one hour per day. Correlation coefficients between the time spent on sports or physical exercise and daily walking were significant but low. The proportions of individuals who did no habitual physical exercise but spent more than one hour per day walking were 46.2% in men and 47.5% in women. The proportion time spent being physically inactive (little physical exercise and little walking) was low in both men and women.

Single-item questions such as those used in this study are often included in epidemiologic and public health questionnaires designed to measure health status and exposure to risk. Measuring physical activity with single-item questions has advantages with regards to the time and money required to obtain data. However, these questions have limitations. Iwai et al.^5^ examined the validity and test-retest reliability of Question 1 and 2 of the JACC Study baseline survey. They suggested that measuring physical activity levels with single-item questions might be appropriate for establishing baseline data that reflects long-term physical activity in large-scale cohort studies targeting lifestyle-related diseases. Tsubono et al.^6^ assessed the validity of Question 2 (daily walking time) using pedometer counts as the reference standard. The geometric mean numbers of walking steps per day measured by the pedometer showed a significant linear association with the responses to Question 2 after adjusting for sex and age.

In conclusion, the proportion of habitual physical exercise was relatively low in the JACC Study and increased with age among participants aged 50-79, whereas almost half walked for more than one hour a day.

## MEMBER LIST OF THE JACC STUDY GROUP

The present investigators involved, with the co-authorship of this paper, in the JACC Study and their affiliations are as follows: Dr. Akiko Tamakoshi (present chairman of the study group), Nagoya University Graduate School of Medicine; Dr. Mitsuru Mori, Sapporo Medical University School of Medicine; Dr. Yutaka Motohashi, Akita University School of Medicine; Dr. Ichiro Tsuji, Tohoku University Graduate School of Medicine; Dr. Yosikazu Nakamura, Jichi Medical School; Dr. Hiroyasu Iso, Institute of Community Medicine, University of Tsukuba; Dr. Haruo Mikami, Chiba Cancer Center; Dr. Yutaka Inaba, Juntendo University School of Medicine; Dr. Yoshiharu Hoshiyama, University of Human Arts and Sciences; Dr. Hiroshi Suzuki, Niigata University School of Medicine; Dr. Hiroyuki Shimizu, Gifu University School of Medicine; Dr. Hideaki Toyoshima, Nagoya University Graduate School of Medicine; Dr. Kenji Wakai, Aichi Cancer Center Research Institute; Dr. Shinkan Tokudome, Nagoya City University Graduate School of Medical Sciences; Dr. Yoshinori Ito, Fujita Health University School of Health Sciences; Dr. Shuji Hashimoto, Fujita Health University School of Medicine; Dr. Shogo Kikuchi, Aichi Medical University School of Medicine; Dr. Akio Koizumi, Graduate School of Medicine and Faculty of Medicine, Kyoto University; Dr. Takashi Kawamura, Kyoto University Center for Student Health; Dr. Yoshiyuki Watanabe, Kyoto Prefectural University of Medicine Graduate School of Medical Science; Dr. Tsuneharu Miki, Graduate School of Medical Science, Kyoto Prefectural University of Medicine; Dr. Chigusa Date, Faculty of Human Environmental Sciences, Mukogawa Women’s University ; Dr. Kiyomi Sakata, Wakayama Medical University; Dr. Takayuki Nose, Tottori University Faculty of Medicine; Dr. Norihiko Hayakawa, Research Institute for Radiation Biology and Medicine, Hiroshima University; Dr. Takesumi Yoshimura, Fukuoka Institute of Health and Environmental Sciences; Dr. Akira Shibata, Kurume University School of Medicine; Dr. Naoyuki Okamoto, Kanagawa Cancer Center; Dr. Hideo Shio, Moriyama Municipal Hospital; Dr. Yoshiyuki Ohno, Asahi Rosai Hospital; Dr. Tomoyuki Kitagawa, Cancer Institute of the Japanese Foundation for Cancer Research; Dr. Toshio Kuroki, Gifu University; and Dr. Kazuo Tajima, Aichi Cancer Center Research Institute.
